# ESTES recommendations for the treatment of polytrauma—a European consensus based on the German S3 guidelines for the treatment of patients with severe/multiple injuries

**DOI:** 10.1007/s00068-025-02852-4

**Published:** 2025-04-11

**Authors:** Cristina Rey Valcarcel, Dan Bieler, Gary A. Bass, Christine Gaarder, Frank Hildebrand

**Affiliations:** 1 Unit of Trauma and Emergency Surgery, Hospital General Universitario Gregorio Marañón, Madrid, Spain; 2 Department for Trauma Surgery and Orthopaedics, Reconstructive and Hand Surgery, Burn Medicine, Germany Armed Forces Central Hospital Koblenz, Rübenacher Strasse, 56072 Koblenz, Germany; 3 Division of Traumatology, Surgical Critical Care and Emergency Surgery, University of Pennsylvania, Philadelphia, PA USA; 4 Department of Traumatology, Oslo University Hospital, Oslo, Norway; 5 Department of Orthopaedics, Trauma and Reconstructive Surgery, University Hospital RWTH Aachen, Aachen, Germany

**Keywords:** Polytrauma, Injury, Guidelines, Recommendations, Consensus

## Abstract

**Introduction:**

Considerable heterogeneity exists in the configuration and implementation maturity of trauma systems across European healthcare settings, and the opportunities for guideline-informed high-quality care varies considerably. Therefore, the European Society of Trauma and Emergency Surgery (ESTES), with its constituent national societies, has developed comprehensive consensus recommendations for care-context appropriate treatment of polytrauma patients in Europe, from the pre-hospital setting to the first surgical phase.

**Methods:**

Adhering to the RAND/UCLA Appropriateness Method (RAM), ESTES conducted a three-round modified Delphi consensus. National society expert delegates assessed Grade of Recommendation (GoR) A and Good Clinical Practice Points (GPP) elements of the German Society of Trauma Surgery (DGU) “*S3 guidelines for polytrauma/severe injury management”* for appropriateness and implementability within their respective healthcare systems.

**Results:**

In the first consensus round, 82 GoR A and 57 GPP recommendations were analysed. Of these, seven GPP were rephrased for clarity and four were removed due to redundancy or conflicting content. Consequently, 135 recommendations (82 GoR A and 53 GPP) remained, with 128 (77 GoR A and 51 GPP) deemed appropriate and necessary, and seven as uncertain due to expert disagreement.

**Conclusion:**

These ESTES recommendations constitute the first cohesive Europe-wide framework for managing the polytrauma patient from the prehospital setting to the end of the first surgical phase. They serve as a foundational tool for the development of national guidelines, particularly in regions with evolving trauma systems, and promote alignment towards a uniform standard-of-care across Europe.

## Introduction

Trauma persists as the foremost cause of death and disability among Europeans under 40 years of age, with accidents and assaults contributing to 3.2% of all recorded deaths in the European Union (EU) in 2021 [1]. Despite its clinical and societal significance, Europe lacks both a unifying healthcare agency and standardized quality assurance programs, thereby perpetuating substantial disparities in trauma system organization and delivery. These deficiencies undermine efforts to establish a common, high-quality standard of trauma care across the EU. Moreover, they impair preparedness for future challenges posed by military, climatic and socio-political crises.

The beneficial impact of comprehensive trauma systems on patient outcomes is well-documented, especially for those with the most severe injuries. Beyond robust personnel and infrastructure, the success of a trauma system hinges on well-coordinated treatment pathways and the application of evidence-based guidance from prehospital care through rehabilitation. This principle is particularly important in the early postinjury phase, where timely intervention can be life-saving [2, 3]^.^

Nevertheless, the implementation and effectiveness of trauma systems exhibit marked variability among European nations [4–6]. In some regions, coordinated trauma care remains insufficiently developed. This reality may also underlie the scarcity of shared treatment guidelines, which, if harmonized, could help ensure uniformly high standards of trauma care across Europe [7].

The European Society of Trauma and Emergency Surgery (ESTES)—comprising experts from thirty-three national societies across 27 countries—offers a uniquely qualified platform for forging consensus on trauma care. Bringing together diverse perspectives, ESTES is positioned to bridge geographic, organizational, and practice-related discrepancies, thereby improving the overall quality of European trauma care.

Building on the German Society of Trauma Surgery (DGU) “S3 guidelines for polytrauma/severe injury management”, we sought to formulate a European consensus on polytrauma management, extending from prehospital care through the initial surgical phase, under the auspices of ESTES and its affiliated national societies of trauma and emergency surgery. As part of this endeavor, we also examined local factors that could impede implementation of certain evidence-based recommendations, aiming to tailor guidelines that accommodate regional nuances while promoting a unified standard of care across Europe.

## Methods

Our study deployed the RAND/UCLA Appropriateness Method (RAM), developed to synthesize existing evidence and obtain the clinical judgement of medical experts on the appropriate treatment of specific clinical presentations [8]. Although often called a"consensus method", RAM does not really belong in that category, because its objective is to detect rather than to obtain consensus. Following a literature review to synthesize the most recent relevant evidence on the procedures to be rated (done within the consensus process of the German S3 guidelines), the expert panel is selected and a two-round modified Delphi Consensus appropriateness rating process is undertaken, followed by a final necessity-rating round for the statements categorized as appropriate [9].

### S3-Guideline on the treatment of polytrauma/severe injuries

The “S3-Guideline on the Treatment of Polytrauma/Severe Injuries” (Polytrauma Guideline), published in December 2022, was developed by 26 German specialist societies under the umbrella of the German Society of Trauma Surgery (DGU) [10]. This guideline provides 332 recommendations for the systematic interdisciplinary care of multiply or severely injured patients spanning the pre-hospital setting, the emergency department and first operative phase. S3 designation, the highest level of quality for guideline development recognized by the German Medical Center for Quality in Medicine (AWMF), requires systematic literature search, evaluation of references, classification of studies and synthesis of recommendations, leading to formal consensus attainment.

We hypothesized that the DGU S3 Polytrauma Guideline recommendations were broadly generalizable across various European healthcare contexts. Each recommendation was graded based on the quality of available evidence and the strength of consensus regarding its benefit-risk ratio, consistency of study results, feasibility, and considerations of economic, legal, ethical, and patient preferences. Three grades of recommendation (GoR) A, B, and 0 were assessed. Additionally, good clinical practice points (GPP) were formulated when, in the absence of robust evidence, the recommendation is supported by a strong clinical consensus on prevailing clinical standard-of-care [11]. To simplify the consensus process, GoR A (30 on pre-hospital care, 36 on emergency department care and 16 on primary operative management) and GPP (25 on pre-hospital care, 18 on emergency department care and 14 on primary operative management) recommendations applicable in all European settings were selected.

### Panel of experts and moderators

**Fig. 1:**
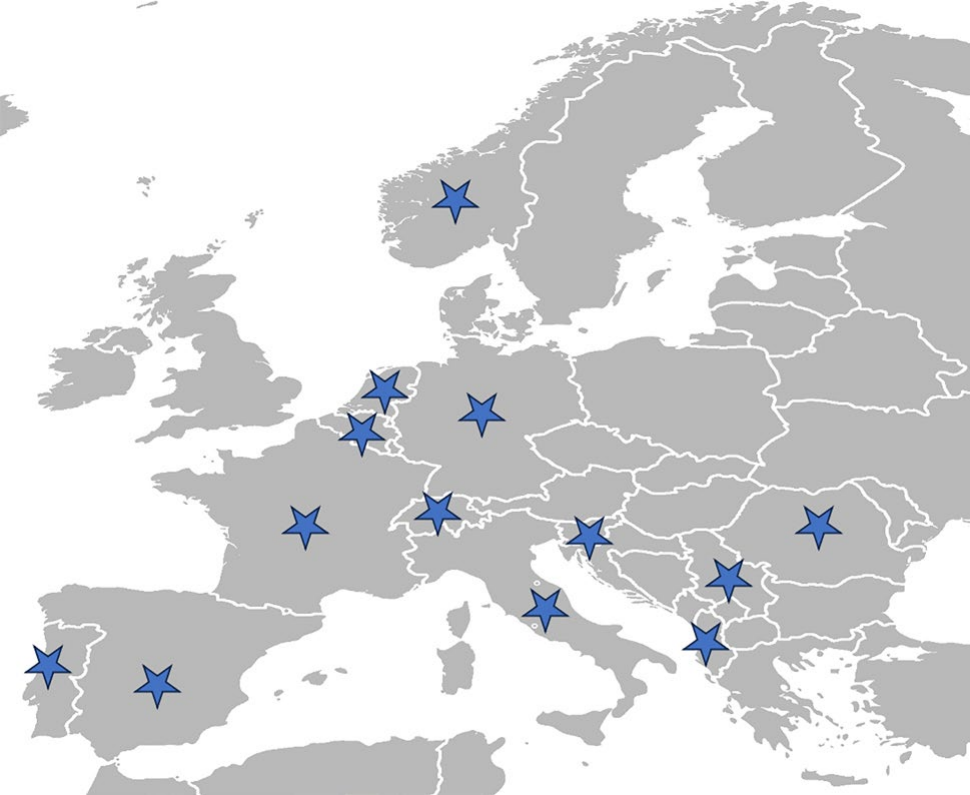
Countries with their national societies involved in the consensus

From the 33 institutional ESTES members, 15 agreed to participate and endorse the consensus (Table 1), representing a total of 13 European countries (Fig. 1). Each society designated a national delegate supported by a local group of experts. In the case of the two participating Portuguese societies, a national panel of experts worked on the project, represented by a single delegate. A dedicated consensus working group (CWG) was constituted with representatives from the ESTES board, sections, and research committee and a DGU delegate involved in the German S3 Guideline development. The CWG moderated the consensus rounds as well as analyzed its results and discrepancies. All participating experts and CWG members completed and signed a declaration of interest document.

**Table 1:** National societies involved in the consensus process

Participating European societies
Albanian Society of Trauma and Emergency Surgery
Belgian Orthopedic Trauma Association
Belgian Trauma Society
Dutch Trauma Society
French Emergency Surgery Society
German Society of Trauma Surgery
Italian Trauma and Emergency Surgery Society
Lusitanian Association of Trauma and Emergency Surgery
Norwegian National Advisory Unit for Trauma
Portuguese Surgical Society
Romanian Society for Trauma and Emergency Surgery
Serbian Society of Emergency Surgery
Slovenian Society of Trauma Surgeons
Spanish Surgeons Association
Swiss Trauma Society

### Appropriateness rating process

The RAM-concept of appropriateness refers to the relative weight of the benefits and harms of a medical or surgical intervention rated in an ordinal quantitative scale (1–9) in a two-round modified Delphi consensus. Delegates first rated the appropriateness of each statement, additionally they were asked about the applicability of the recommendation in their local practice to understand possible disagreements. These first results were analysed by the CWG, focusing on disagreements. If rating discrepancies were caused by regional particularities that prevent the application of the recommendation, the CWG contacted the national delegate to discuss the regional particularity. After this process, GPP recommendations were either rephrased, if translation or minor applicability issues were detected, or cancelled, if they were considered redundant or not applicable for most countries. The CWG elected not to change GoR A recommendations as these were formulated according to the existing evidence at the German consensus.

The modified list of recommendations, with median rates of the first round, was sent to the expert panel one month before rating in a web-based meeting. At this second round performed in July 2024, panelists discussed the recommendations, particularly focusing on those with disagreement. If desired, the opportunity to modify the list of indications and/or definitions within the recommendation was discussed. After discussing each chapter of the list of recommendations, each single recommendation was re-rated. No attempt was made to force the panel to consensus.

At the final analysis each statement was classified as “appropriate,” “uncertain” or “inappropriate” in accordance with the panelists’ median score and the level of disagreement among the panelists. Statements with median scores in the 1–3 range were classified as inappropriate, those in the 4–6 range as uncertain, and those in the 7–9 range as appropriate. However, all indications rated “with disagreement,” whatever the median, were classified as uncertain. We used a strict criterion to define “Disagreement” or lack of consensus, either because there was polarization of the group or because judgements were spread over the entire 1–9 rating scale.

### Necessity rating process

The concept of necessity represents a stricter criterion than appropriateness. When a procedure is necessary, its expected benefits exceed the potential negative consequences by such that the procedure must be offered by the health care system. A recommendation should be considered necessary when meeting all the following criteria: The statement is appropriate, it would be considered improper care not to provide this service, there is a reasonable chance that this procedure will benefit the patient and the benefit to the patient is not small. At the necessity rating round the expert panel rate each appropriate recommendation individually and anonymously, in a scale of 1–9 (1 meaning that the procedure is clearly not necessary and 9 that it is clearly necessary). A recommendation was considered necessary if the median rate of experts was ≥ 8.

## Results

**Fig. 2:**
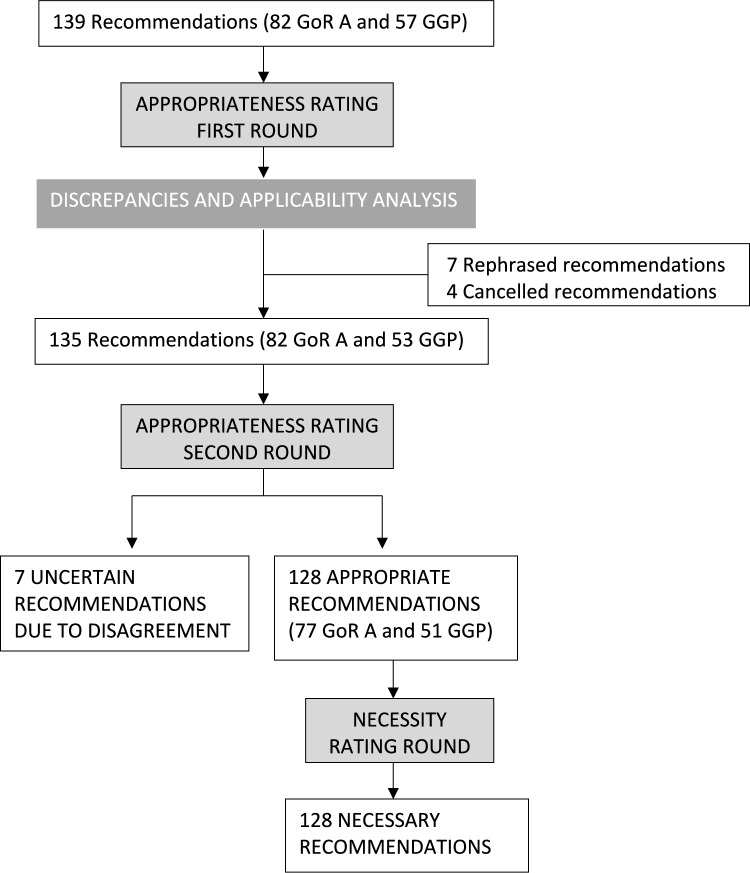
Consensus flow chart

The first round of the appropriateness consensus was conducted in January and February 2024. The panel of experts rated the original 139 GoR A and GPP S3-Guideline recommendations of the German Society of Trauma Surgery. After the first analysis, 23 recommendations were categorized as uncertain due to disagreement and therefore investigated by the CWG looking for local discrepancies, applicability and translation issues that might prevent the agreement on these recommendations. As a result, seven GPP recommendations were rephrased to improve their meaning and local applicability, and four GPPs were omitted for redundancy or information incongruency (Fig. 2).

At the second round, 135 recommendations were rated by the panel of experts during a web-based meeting. In the final consensus analysis, 128 recommendations (77 GoR A and 51 GPP) were categorized as appropriate (Table 2) and seven as uncertain due to disagreement (Table 3). All the recommendations categorized as appropriate by the panel of experts were considered necessary at the necessity rating round conducted in September 2024.

**Table 2:** Recommendations categorized as appropriate and necessary

Statements categorized as appropriate and necessARY	Approp. median rate (RIQ)	Approp. categorization (n = 14)			Necessity categorization,median rate	LoE DGOU
		Ap	Un	Inap	(RIQ)	
*1.Prehospital care*						
1.1 Stop the bleeding						
1.1.1: Active bleeding should always be stopped if it is amenable to hemostasis in the pre-hospital setting	9 (9–9)	14	0	0	Necessary 9(9–9)	GGP
1.1.2: Patients with clinical evidence of pelvic ring injury or unstable pelvic ring injury and hemodynamic instability should receive a pelvic binder	9 (9–8)	13	1	0	Necessary 9(9–9)	GGP
1.1.3: Active bleeding of the extremities should be treated according to the following stage regime: (1) Manual compression (2) Compression bandage, if possible, in combination with hemostatic agents (3) Tourniquet	9(9–9)	14	0	0	Necessary 9(9–9)	A↑↑
1.1.4: If there are other options for bleeding control, then the manual compression, even though sufficient, can be abandoned in favor of the other procedure. When doing manual compression repetitive checks to see if bleeding has stopped should not be done	9(9–7)	11	3	0	Necessary 9(9–8)	GGP
1.1.5: A tourniquet should be used when life threatening bleeding cannot be stopped in a timely manner by other measures	9(9–9)	13	1	0	Necessary 9(9–9)	A ↑↑
1.1.6: If a tourniquet has been applied for proximal control of inaccessible but compressible hemorrhage, reassess its need and consider alternative treatments when hemodynamically appropriate, with reference to the recorded duration of ischemia *	9(9–7)	13	1	0	Necessary 9(9–7)	GGP
1.2 Air way management, ventilation and emergency anesthesia						
1.2.1: For multiply injured patients with apnea or agonal breathing (respiration rate < 6), emergency anesthesia, endotracheal intubation and ventilation must be performed in the prehospital setting	9(9–9)	14	0	0	Necessary 9(9–9)	A ↑↑
1.2.2: Hemodynamic changes during the pre-hospital or in-hospital induction of anesthesia should be monitored closely and treated early if necessary	9(9–9)	14	0	0	Necessary 9(9–9)	GGP
1.2.3: Multiply injured patients must be pre-oxygenated before anesthesia induction	9(9–9)	14	0	0	Necessary 9(9–9)	A ↑↑
1.2.4: Emergency personnel must be regularly trained in performance and assistance with emergency anesthesia, endotracheal intubation, and alternative methods of airway protection (mask ventilation, laryngeal tube, cricothyrotomy)	9(9–8)	14	0	0	Necessary 9(9–9)	A ↑↑
1.2.5: A difficult airway must be anticipated when performing endotracheal intubation of the trauma patient	9(9–9)	14	0	0	Necessary 9(9–9)	A ↑↑
1.2.6: During anesthesia induction and endotracheal intubation of the polytrauma patient, alternative methods to secure the airway must be available	9(9–9)	14	0	0	Necessary 9(9–9)	A ↑↑
1.2.7: Alternative methods of ventilation and/or securing the airway must be considered after more than 2 attempts at intubation	9(9–9)	14	0	0	Necessary 9(9–9)	A ↑↑
1.2.8: ECG, blood pressure, pulse oximetry and capnography must be monitored during anesthesia induction, endotracheal intubation, ventilation, and anesthesia	9(9–9)	14	0	0	Necessary 9(9–9)	A ↑↑
1.2.9: The capnometry/capnography must be used pre-hospital and in-hospital during endotracheal intubation to control tube placement and afterwards, to monitor displacement and ventilation	9(9–9)	14	0	0	Necessary 9(9–9)	A ↑↑
1.2.10: Normoventilation must be carried out for endotracheally intubated and anesthetized trauma patients	9(9–9)	14	0	0	Necessary 9(9–9)	A ↑↑
1.2.11: Beginning in the Emergency Department, ventilation must be monitored and controlled with frequent arterial blood gas analysis	9(9–9)	14	0	0	Necessary 9(9–7)	A ↑↑
1.2.12: Emergency anesthesia for endotracheal intubation must be performed with rapid sequence induction due to aspiration risk of polytrauma patients	9(9–9)	14	0	0	Necessary 9(9–9)	A ↑↑
1.2.13: A pre-hospital induced extra glottic airway should be immediately transferred to endotracheal intubation in the hospital using video laryngoscopy	9(9–8)	13	1	0	Necessary 9(9–7)	GGP
1.2.14: Coniotomy should be performed using surgical technique. If there is a special level of practice with another coniotomy technique, this can be used	9(9–9)	13	1	0	Necessary 9(9–9)	GGP
1.2.15: A guide rod or “bougie “ should be used whenever the polytraumatized patient is intubated by video laryngoscopy	8(9–7)	11	3	0	Necessary 9(9–8)	GGP
1.3 Coagulation management and volume replacement						
1.3.1: Intravenous access must be placed in trauma patients	9(9–9)	13	1	0	Necessary 9(9–9)	A ↑↑
1.3.2: In trauma patients where venous access cannot be achieved, establish an intraosseous access for infusion and medication therapy	9(9–9)	14	0	0	Necessary 9(9–9)	A ↑↑
1.3.3: In case an adequate blood pressure is not achieved in the polytraumatized patient by sufficient volume therapy, titrating vasopressors may be considered for circulatory support	9(9–9)	14	0	0	Necessary 9(9–8)	GGP
1.3.4: The lethal triad, hypothermia, acidosis, and coagulopathy should be already counteracted pre-hospital by: 1. Prevention of further cooling of the patient (aim: normothermia) 2. Appropriate therapy of hemorrhagic shock (hemorrhage control, volume, and coagulation therapy) 3. Adequate oxygenation and ventilation (if necessary, intubation according to intubation criteria)	9(9–9)	14	0	0	Necessary 9(9–9)	GGP
1.4 Analgesia						
1.4.1: Severely injured patients should receive intravenous analgesia	9 (9–9)	12	1	0	Necessary 9(9–8)	A↑↑
1.4.2: Responsive severely injured patients should be considered and eventually asked for pain medication*	9 (9–9)	14	0	0	Necessary 9(9–9)	GGP
1.4.3: The Numeric Rating Scale is not applicable in all patients, alternatively patients should be asked for subjective level pain	9 (9–7)	11	3	0	Necessary 8(9–7)	GGP
1.4.4: The Numeric Rating Scale should be used to objectify and to document the pain as well as to control the success of analgesia	9 (9–7)	12	2	0	Necessary 8,5(9–7)	A↑↑
1.4.5: Target value of the analgesia should be a Numeric Rating Scale ≤ 4	9 (9–7)	11	3	0	Necessary 8,5(9–7)	A↑↑
1.4.6: In addition to pharmacological therapy, physical measures (e.g., positioning, splinting) should be used	9 (9–9)	14	0	0	Necessary 9(9–9)	GGP
1.4.7: Analgesia should be administered, after appropriate education and training, with continuous monitoring (e.g., ECG, blood pressure, respiratory and heart rate, pulse oximetric oxygen saturation and capnography, if necessary) of the patient. Emergency equipment should be readily available to treat complications	9 (9–9)	14	0	0	Necessary 9(9–9)	GGP
1.5 Thorax						
1.5.1: Clinical examination of the chest and respiratory function must be performed	9(9–9)	14	0	0	Necessary 9(9–9)	A↑↑
1.5.2: A provisional diagnosis of pneumothorax and/or hemothorax should be made in cases of unilateral absence or decrease in breath sounds (after controlling for correct tube placement) or when there are sonographic signs	9(9–9)	14	0	0	Necessary 9(9–9)	A↑↑
1.5.3: Clinically suspected tension pneumothorax must be decompressed immediately	9(9–9)	14	0	0	Necessary 9(9–9)	A↑↑
1.5.4: Open pneumothorax should be treated with a suitable valve dressing	9(9–9)	14	0	0	Necessary 9(9–9)	GGP
1.6 Traumatic brain injury						
1.6.1: Repeated examinations and documentation of level of consciousness, pupillary function and Glasgow Coma Scale must be performed	9(9–9)	14	0	0	Necessary 9(9–9)	A↑↑
1.6.2: Glucocorticoid administration must be avoided	9(9–9)	14	0	0	Necessary 9(9–9)	A↑↑
1.6.3: Avulsed teeth and tooth fragments should be collected, stored in a moist environment accompanying the patient *	9(9–8)	14	0	0	Necessary 9(9–7)	GGP
1.7 Spine						
1.7.1: A targeted physical examination including the spine and related functions must be performed	9(9–9)	14	0	0	Necessary 9(9–9)	A↑↑
1.7.2: In unconscious patients, spine injury must be assumed until there is evidence to exclude it	9(9–9)	14	0	0	Necessary 9(9–9)	A↑↑
1.7.3: The cervical spine must be immobilized prior to the actual technical rescue during rapid and careful rescues. An exception is the need for immediate rescue (e.g., fire or risk of explosion	9(9–9)	13	1	0	Necessary 9(9–9)	GGP
1.7.4: Transport should be as gentle and pain-free as possible	9(9–9)	14	0	0	Necessary 9(9–9)	GGP
1.8 EXTREMITIES						
1.8.1: Profusely bleeding extremity injuries that can impair vital functions must be given priority	9 (9–9)	14	0	0	Necessary 9(9–9)	A↑↑
1.8.2: The treatment of extremity injuries must avoid further damage and not delay overall rescue time in cases when other threating injuries are present	9(9–9)	14	0	0	Necessary 9(9–9)	A↑↑
1.9 Transport and target hopsital						
1.9.1: In cases of penetrating thoracic or abdominal trauma, the patient should be transported as quickly as possible to the nearest trauma capable facility *	9(9–9)	13	1	0	Necessary 9(9–9)	GGP
1.9.2: To avoid transition problems during registration and/or transfer of severely injured patients, appropriate and standardized communication methods must be used	9(9–9)	14	0	0	Necessary 9(9–9)	GGP
1.10 Mass casualty incident						
1.10.1: A hospital alert and response plan should be drawn up by each hospital, implemented in their own facility and regularly evaluated through exercises	9(9–8)	12	2	0	Necessary 9(9–9)	GGP
1.10.2: The preparation of responsible physicians for a (terror) mass casualty incident scenario should through regular exercises	9(9–9)	13	1	0	Necessary 9(9–9)	GGP
*2. Emergency department*						
2.1 Emergency department-trauma team and activation						
2.1.1: For care of polytrauma patients, a specific team (the “Trauma Team”) must work according to an organized plan and/or have completed special training	9(9–9)	13	1	0	Necessary 9(9–9)	A↑↑
2.1.2: The interprofessional trauma team must consist of at least 2 nurses and at least 2 physicians who represent emergency medicine and emergency surgery expertise	9(9–9)	13	1	0	Necessary 9(9–9)	GGP
2.1.3: Trauma centers must keep expanded trauma teams at any time according to the level of care of the hospital	9(9–9)	14	0	0	Necessary 9(9–9)	GGP
2.1.4: Trauma team should be activated in the event of the following pathologic findings after trauma: A/B—Problem Respiratory distress (SpO2 < 90%)/required airway protection Respiratory rate < 10 or > 29 C—Problem Systolic blood pressure < 90 mmHg Heart rate > 120/min Shock index > 0,9 Positive eFAST D- Problem GCS ≤ 12 E—Problem Hypothermia < 35,0 °C	9 (9–9)	13	1	0	Necessary 9(9–9)	A↑↑
2.1.5: The Emergency Department Trauma Team should be activated in the event of the following injuries or actions following trauma: Unstable thorax Mechanically unstable pelvic injury Presence of penetrating injury to the trunk-neck-region Amputation injury proximal to the hands/feet Sensorimotor deficit following spinal injury Pre-hospital intervention (required airway protection, thoracic decompression, catecholamine administration, pericardiocentesis or tourniquet application)	9(9–9)	13	1	0	Necessary 9(9–9)	A↑↑
2.2 Resuscitation						
2.2.1: In case of unconsciousness and no breathing or agonal breathing cardiopulmonary resuscitation procedures should be started immediately	9(9–9)	12	2	0	Necessary 9(9–9)	A↑↑
2.2.2: For the treatment of trauma induced cardiac arrest, it must be underscored that the pathophysiology is different form non-traumatic cardiac arrest, and thus the procedure is fundamentally different	9(9–9)	13	1	0	Necessary 9(9–9)	A↑↑
2.2.3: Resuscitation of trauma-induced cardiovascular arrest should focus on the immediate, simultaneous treatment of potentially reversible causes and takes precedence over chest compression	9(9–9)	13	1	0	Necessary 9(9–9)	GGP
2.2.4: During cardiopulmonary resuscitation, the trauma specific, reversible causes of cardiac arrest (according to the ABCDE protocol, e.g., external bleeding, airway obstruction, esophageal intubation, tension pneumothorax, pericardial tamponade, and hypovolemia) must be diagnosed, excluded and/or treated	9(9–9)	14	0	0	Necessary 9(9–9)	A↑↑
2.2.5: In trauma associated cardiovascular arrest, a sequential approach should be adopted with Hemostasis (for massive external bleeding) Airway protection Bilateral pleural space decompression via surgical minithoracotomy Non-invasive external pelvic stabilization Blood products and in certain circumstances Emergency thoracotomy for removal of pericardial tamponade a proximal aortic clamping or REBOA	9(9–9)	14	0	0	Necessary 9(9–9)	GGP
2.2.6: In trauma associated cardiovascular arrest all measures (e.g., external pressure, hemostatic agents and Tourniquets, pelvic sheeting) should be performed to control bleeding	9(9–9)	14	0	0	Necessary 9(9–9)	GGP
2.2.7: If tension pneumothorax is suspected, bilateral relief by minithoracotomy should be performed in patients with trauma induced cardiovascular arrest	9(9–9)	14	0	0	Necessary 9(9–9)	A↑↑
2.2.8: An in-hospital emergency thoracotomy should be used for the following indications (pre-hospital resuscitation < 10 min, cardiovascular arrest in the shock room) in the setting of observed cardiovascular arrest in the trauma patient	9(9–7)	12	2	0	Necessary 9(9–9)	GGP
2.2.9: Before cessation of resuscitation measures, all potentially reversible causes of trauma-induced cardiac arrest must be excluded or treated	9(9–9)	14	0	0	Necessary 9(9–9)	GGP
2.2.10: When resuscitation has failed after eliminating all possible trauma specific, reversible causes of cardiac arrest, cardiopulmonary resuscitation must be ended	9(9–9)	13	1	0	Necessary 9(9–9)	A↑↑
2.2.11: If there are clear signs of death, or injuries incompatible with life, cardiopulmonary resuscitation must not be started	9(9–9)	14	0	0	Necessary 9(9–9)	A↑↑
2.3 Coagulation management and volume replacement						
2.3.1: Trauma-induced coagulopathy is an independent clinical picture with clear effects on survival. Thus, coagulation assessment and therapy must be begun at the latest in the emergency department	9(9–9)	13	1	0	Necessary 9(9–9)	A↑↑
2.3.2: Basic laboratory assessments of severe trauma with hemorrhage must include early and repeated measurement of blood gas analysis, PT, aPTT, fibrinogen, and platelet count as well as blood type determination	9(9–9)	13	1	0	Necessary 9(9–9)	A↑↑
2.3.3: When treating severely injured and bleeding patients in the emergency department, in addition to other diagnostic studies and therapy for trauma-induced coagulopathy, perform early viscoelastic testing	9(9–7)	12	2	0	Necessary 9(9–7)	A↑↑
2.3.4: The extent of shock as well as the treatment of shock must be monitored and controlled with repeated measurements of base excess and/or lactate levels	9(9–9)	14	0	0	Necessary 9(9–9)	A↑↑
2.3.5: Guide coagulation diagnostics and therapy by viscoelastic testing procedures	9(9–7)	11	3	0	Necessary 9(9–7)	A↑↑
2.3.6: For profusely bleeding patients, tranexamic acid (TxA) must be administered as soon as possible/in the pre-hospital *	9(9–9)	13	1	0	Necessary 9(9–9)	A↑↑
2.3.7: Within 24 h of hemostasis, decisions must be made regarding the type and beginning of thromboprophylaxis	9(9–9)	13	1	0	Necessary 9(9–9)	GGP
2.3.8: The creation of a central vascular access site should be ultrasound-guided, if immediately available	9(9–7)	12	2	0	Necessary 8(9–7)	GGP
2.4 Imaging						
2.4.1: If it is unclear whether there is a relevant pelvic injury and CT is not performed immediately, pelvic X-rays may be done *	9(9–9)	14	0	0	Necessary 9(9–9)	GGP
2.4.2: In the context of diagnostic studies for severely injured patients, whole-body CT ((Head to and including pelvis, CCT without contrast) with a trauma-specific protocol must be performed in a timely manner, if there is no immediate need for intervention/surgery and/or resuscitation and the SBP > 60 mmHg	9(9–9)	13	1	0	Necessary 9(9–7)	A↑↑
2.4.3: Magnetic resonance imaging may be indicated for specific questions (e.g., disco ligamentous spinal injuries, morphological correlate of paraplegic symptoms) in further primary diagnostics. Extensive requirements must be met for the performance of MRI as spart of the initial diagnosis of severely injured/polytraumatized patients. Corresponding specifications should be available in SOPs for each location	9(9–8)	14	0	0	Necessary 9(9–7)	GGP
2.5 Interventional control of hemorrhage or vascular injuries						
2.5.1: Consider endovascular control of vascular injury in hemodynamically stabilized patients, where local expertise and facilities are available *	9(9–8)	14	0	0	Necessary 9(9–9)	GGP
2.6 Thorax						
2.6.1: Clinical examination of the chest must be performed	9(9–9)	14	0	0	Necessary 9(9–9)	A↑↑
2.6.2: Auscultation must be carried out with the physical examination	9(9–7)	11	3	0	Necessary 9(9–9)	A↑↑
2.6.3: Continuous monitory by a 3-lead ECG must be performed to monitor vital functions/any myocardial damage	9(9–9)	14	0	0	Necessary 9(9–9)	A↑↑
2.6.4: If blunt cardiac injury is suspected, a twelve lead ECG in combination with the determination of serum levels of HS Troponin in the blood should be performed	9(9–9)	12	2	0	Necessary 9(9–9)	A↑↑
2.6.5: Clinically relevant or progressive pneumothorax must be primarily decompressed by chest tube drainage in ventilated patients	9(9–9)	14	0	0	Necessary 9(9–9)	A↑↑
2.6.6: For hemodynamically unstable patients with thoracic trauma, an eFAST examination should be performed to rule out pericardial tamponade	9(9–9)	14	0	0	Necessary 9(9–9)	GGP
2.6.7: Thoracotomy can be performed with initially high blood loss or continuing blood loss from the chest tube in both stable as well as unstable patients	9(9–9)	12	2	0	Necessary 9(9–9)	GGP
2.6.8: As an alternative for thoracotomy VATS (video-assisted thoracoscopy) can be performed in cardio-pulmonal stable patients	9(9–8)	14	0	0	Necessary 9(9–8)	GGP
2.7 Abdomen						
2.7.1: The abdomen must be examined, although an unremarkable examination does not exclude a relevant intraabdominal injury, even in conscious patients	9(9–9)	14	0	0	Necessary 9(9–9)	A↑↑
2.7.2: Multi-slice spiral CT (MSCT) has high sensitivity and the highest specificity for detecting intra-abdominal injuries and therefore must be performed after abdominal trauma	9(9–9)	14	0	0	Necessary 9(9–9)	A↑↑
2.8 Pelvis						
2.8.1: Acute life-threatening pelvic injury must be excluded upon patient arrival to the hospital	9(9–9)	14	0	0	Necessary 9(9–9)	A↑↑
2.8.2: If bone injury of the pelvis is suspected pelvic radiographs or computer tomography (CT) should be performed	9(9–9)	14	0	0	Necessary 9(9–9)	A↑↑
2.8.3: In cases of a mechanically unstable pelvic ring and hemodynamic instability, emergency mechanical stabilization of the pelvis must be carried out	9(9–9)	14	0	0	Necessary 9(9–9)	A↑↑
2.9 Traumatic brain injury						
2.9.1: Examinations of level of consciousness (with pupillary function) and GCS (bilateral motor function) must be repeated at regular intervals and documented	9(9–9)	14	0	0	Necessary 9(9–9)	A↑↑
2.9.2: The goals should be normal oxygen, carbon dioxide, and blood pressure levels. Arterial oxygen saturation falling below 90% must be avoided	9(9–9)	14	0	0	Necessary 9(9–9)	A↑↑
2.9.3: Unconscious patients (GCS ≤ 8) must be intubated with adequate ventilation (according to capnometry and blood gas analysis)	9(9–9)	13	1	0	Necessary 9(9–9)	A↑↑
2.9.4: In adults, the goal should be normal arterial pressure with SBP > = 110 mmHg	9(9–9)	14	0	0	Necessary 9(9–9)	A↑↑
2.9.5: In cases of polytrauma with suspected traumatic brain injury, head CT must be performed	9(9–9)	14	0	0	Necessary 9(9–9)	A↑↑
2.9.6: In cases of neurological deterioration, a control CT must be performed	9(9–9)	14	0	0	Necessary 9(9–9)	A↑↑
2.9.7: For treatment of TBI, glucocorticoid administration must be avoided	9(9–9)	14	0	0	Necessary 9(9–9)	A↑↑
2.10 Neck						
2.10.1: Securing the airway must take priority when treating neck injuries	9(9–9)	14	0	0	Necessary 9(9–9)	A↑↑
*3 Primary operative management*						
3.1 Thorax						
3.1.1: In the cardiorespiratory stable patients, VATS (video-assisted thoracoscopy) can be used if surgery is needed	9(9–9)	12	2	0	Necessary 8(9–6)	GGP
3.1.2: A penetrating chest injury that is the cause of hemodynamic instability must undergo immediate exploratory thoracotomy	9(9–9)	13	1	0	Necessary 9(9–9)	A↑↑
3.1.3: Thoracotomy can be performed in cases with initially high blood loss or persistent relevant blood loss via the chest tube in both stable and unstable patients	9(9–9)	12	2	0	Necessary 9(9–9)	GGP
3.1.4: As an alternative to the thoracotomy, VATS (video-assisted thoracoscopy) may be performed in cardio-respiratory stable patients	9(9–9)	12	2	0	Necessary 9(9–6)	GGP
3.2 ABDOMEN						
3.2.1: Penetrating colon injuries must be controlled by suturing or resection to reduce the risk of intraabdominal infections	9(9–9)	14	0	0	Necessary 9(9–9)	A↑↑
3.3 Traumatic brain injury						
3.3.1: Space-occupying intracranial injuries must be treated as a surgical emergency	9(9–9)	14	0	0	Necessary 9(9–9)	A↑↑
3.4 Spine						
3.4.1: In the presence of a fracture morphology with spinal canal compression or translational injury, and without the ability to rule out spinal neurological damage, it should be assumed to exist until it can be excluded	9(9–9)	14	0	0	Necessary 9(9–9)	GGP
3.4.2: Initial stabilization should be performed early, taking the overall condition of the patient into account	9(9–9)	14	0	0	Necessary 9(9–9)	GGP
3.4.3: Stabilization at the cervical spine can be performed ventrally and/or dorsally, depending on the injury, or in exceptional cases using a halo fixator	9(9–9)	14	0	0	Necessary 9(9–9)	GGP
3.4.4: For thoracic and lumbar spine injuries, the dorsal internal fixator should be used as the primary operative method for stabilization	9(9–9)	14	0	0	Necessary 9(9–9)	GGP
3.5 Mandible and midface						
3.5.1: In mandibular and maxillofacial injuries, primary airway protection and hemostasis in the oral and maxillofacial area must be carried out	9(9–9)	14	0	0	Necessary 9(9–9)	A↑↑
3.6 Neck						
3.6.1: Provided that intubation or tracheotomy has not yet been performed, clinical findings relating to the airways must be observed and evaluated prior to anesthetic induction for intubation	9(9–9)	14	0	0	Necessary 9(9–9)	A↑↑
3.6.2: Intubation tools and a cricothyrotomy (coniotomy) set must be kept available for immediate use. A ‘‘difficult airway’’ algorithm must be adhered to	9(9–9)	14	0	0	Necessary 9(9–9)	A↑↑
3.6.3: A previously performed cricothyrotomy must be closed operatively. When necessary, tracheotomy must be performed	9(9–9)	14	0	0	Necessary 9(9–9)	A↑↑
3.7 Hand						
3.7.1: During the primary operative phase, dislocations must be reduced and immobilized	9(9–9)	14	0	0	Necessary 9(9–9)	A↑↑
3.7.2: For perilunar dislocation/fracture, reduction must be performed during the primary operative phase, open if necessary	9(9–9)	13	1	0	Necessary 9(9–9)	A↑↑
3.7.3: For decisions regarding replantation, overall injury severity must be considered and the principle ‘‘life before limb’’ must be applied	9(9–9)	14	0	0	Necessary 9(9–9)	A↑↑
3.7.4: The decision to perform time-consuming hand salvage attempts is done on a case-by-case basis. The overall injury severity and the severity of the hand injury must be considered	9(9–9)	14	0	0	Necessary 9(9–9)	A↑↑
3.7.5: For manifest compartment syndrome of the hand, fasciotomy must be performed immediately	9(9–9)	14	0	0	Necessary 9(9–9)	A↑↑
3.8 Lower extremity						
3.8.1: Dislocations of the lower extremity must be reduced and retained at the earliest opportunity	9(9–9)	14	0	0	Necessary 9(9–9)	A↑↑
3.8.2: Perioperative antibiotic prophylaxis must be administered for both open and closed lower extremity fracture surgeries	9(9–9)	14	0	0	Necessary 9(9–9)	A↑↑
3.8.3: In compartment syndrome of the lower extremity, immediate compartment decompression and fixation of a concomitant fracture must be performed	9(9–9)	14	0	0	Necessary 9(9–9)	A↑↑
3.9 Foot						
3.9.1: For a manifest compartment syndrome of the foot, fasciotomy must be performed immediately	9(9–9)	14	0	0	Necessary 9(9–9)	A↑↑
3.10 Thermal skin injuries and burns						
3.10.1: When burns are present in addition to other injuries in a severely injured patient, pre-hospital treatment priorities remain the same	9(9–9)	14	0	0	Necessary 9(9–9)	GGP
3.10.2: In severely injured patients, burn injuries should not be cooled	9(9–9)	14	0	0	Necessary 9(9–9)	GGP
3.10.3: Severely injured patients with burns should be transported to the closest trauma center. When there is equal accessibility, a trauma center specializing in burn injuries is preferable	9(9–9)	14	0	0	Necessary 9(9–9)	GGP
3.10.4: When burns are present in addition to other injuries in a severely injured patient, emergency department treatment priorities remain the same	9(9–9)	14	0	0	Necessary 9(9–9)	GGP
3.10.5: In cases of burns to the torso affecting respiratory mechanics, escharotomy must be performed immediately	9(9–9)	14	0	0	Necessary 9(9–9)	GGP
3.10.6: For burns of the extremities affecting perfusion, rapid escharotomy must be performed	9(9–9)	14	0	0	Necessary 9(9–9)	GGP
3.10.7: Once the vital signs are stabilized and the necessary primary operative management is performed, the severely burned patient must be transferred to a burn center associated with a national trauma center	9(9–9)	14	0	0	Necessary 9(9–9)	GGP

**Table 3:** Recommendations categorized as uncertain

Recommendations categorized as uncertain	Consensus median rate (RIQ)	Consensus categorization (14 National Societies)			LoE DGOU
		Approp	Uncertain	Inapprop	
1.Prehospital care					
1.a: In case of bleeding penetrating injuries in which the foreign body has already been removed and which at least has a length of 3 cm, a direct wound tamponade with chitosan-based hemostatic should be carried out	4 (7–1)	6	2	6	A↑↑
1.b: If there is uncertainty in the assessment of correct tube position by capnography (e.g., in severe shock, hypothermia, CPR or suspected device failure) the tube position should be checked immediately by video laryngoscopy or alternatively by bronchoscopy	8 (9–7)	12	1	1	GGP
1.c: Crystalloids should be used for volume replacement in trauma patients. Balanced, isotonic, crystalloid electrolyte solutions, which are ideally prewarmed, should be used for volume therapy in trauma patients	5 (9–3)	6	3	5	A↑↑
1.d: Fentanyl, ketamine und morphine have a comparable efficacy and should be used for analgesia in spontaneously breathing severely injured patients	8 (9–7)	13	0	1	A↑↑
2. Emergency department					
2.a: The REBOA can be used for temporary proximal hemorrhage control in the setting of trauma induced resuscitation	5(8–1)	4	5	5	GGP
2.b: Patients with life-threatening hemorrhage and/or shock, should receive additional administration of fibrinogen (initially 3–6 g or 30–60 mg/kg)	6(8–3)	7	3	4	A↑↑
2.c: The pelvis must be physically examined	7(9–5)	8	3	3	A↑↑

## Discussion

This is the first joint effort of the National Societies as institutional members of ESTES and ESTES itself to provide European recommendations for the management of the severely-injured patients in the early posttraumatic phase (prehospital setting, emergency department and first surgical approach). Despite, having different trauma system models and great variation on its maturation, we achieved a strong level of expert agreement despite heterogeneity in trauma system configurations and degrees of maturity across the 13 represented European countries. Overall agreement on the GoR A and GPP recommendations for the management of severely injured patients proposed by the S3 Guidelines was greater than 92%, with 128 out of 139 recommendations rated as appropriate and necessary.

None of the recommendations was rated as inappropriate, but seven were rated as uncertain under the strict criterion we used to qualify a disagreement. These recommendations belong to the prehospital and emergency department phases. Disagreement mainly came from different practices, organizational aspects and/or resources across European countries or from new evidence published after the S3 Guidelines publication (December 2022).

### Prehospital care recommendation categorized as uncertain.

Disagreement on all recommendations rated as uncertain in this chapter can be explained by different local practices reflecting the substantial variations in the organization, staffing and resource allocation of emergency medical services (EMS) among the European countries or even regions [6, 12].

1.a. “In case of bleeding penetrating injuries in which the foreign body has already been removed and which at least has a length of 3 cm, a direct wound tamponade with chitosan-based hemostatic should be carried out”.

Pre-hospital use of chitosan-based hemostatic dressings for civilian trauma patients with penetrating injuries is recommended by the S3 Guidelines of the DGU based on a few studies that showed a reduced time to hemostasis and blood lost compared to conventional gauze dressings [13, 14]. However, these adjuncts are not widely available in some European EMS. Furthermore, given the paucity of efficacy or superiority data comparing hemostatic agents and conventional dressings in the trauma setting, the panel of experts recommended the use of either hemostatic or conventional dressings in penetrating injuries, based on physician preference and resource availability.

1.b. “If there is uncertainty in the assessment of correct tube position by capnography (e.g., in severe shock, hypothermia, CPR or suspected device failure) the tube position should be checked immediately by video laryngoscopy or alternatively by bronchoscopy”.

Although all participants at the consensus meeting agreed on the principle of this statement, two delegates considered it inappropriate or uncertain for their national practice, as ultrasonography (US) is the most often used tool to check endotracheal tube position by EMS in their countries. The development of handheld and low-cost devices led US to become widely adopted by EMS worldwide. Recent evidence (sensitivity 98%, specificity 95%) supports sonography, performed by trained EMS staff, for endotracheal tube placement confirmation [15]. Based on these promising results, high availability of US devices and simplicity, some European EMS prefer this technique in their daily practice compared to wave-capnography.

1.c. “Crystalloids should be used for volume replacement in trauma patients. Balanced, isotonic, crystalloid electrolyte solutions, which are ideally prewarmed, should be used for volume therapy in trauma patients.”

All panel experts agreed on the superiority of crystalloids over colloids based on high quality evidence [7], but did not support crystalloids when blood components are available. Early administration of blood components is a cornerstone of hemostatic resuscitation in trauma patients at risk of massive bleeding and shock. Following the first reports that suggested some benefit administrating red blood cells and pre-thawed plasma during military casualty retrieval [16, 17], prehospital transfusion has been increasingly adopted also in the civilian setting. Although randomized trials demonstrated equipoise regarding survival outcomes [18–20], secondary analysis and systematic reviews suggest a benefit in selected patients, especially if transport time to hospital is longer than 20 minutes [21]. Some European countries, mostly in those were geographical characteristics and a low population density led to long transport times to hospital, ensure huge logistical efforts and resources to organize reliable and safe programs to transfuse blood components in the prehospital setting. Delegates from these countries did not consider the use of crystalloids for resuscitation of trauma patients at risk of massive bleeding as appropriate. However, for those delegates coming from mainly urban countries or from European countries without prehospital blood component availability, it is an acceptable recommendation.

1.d. “Fentanyl, ketamine und morphine have a comparable efficacy and should be used for analgesia in spontaneously breathing severely injured patients”.

This statement did not reach full consensus due to the strict criterion we used to define disagreement. The three proposed drugs demonstrated efficacy for analgesia in spontaneously breathing trauma patients [22] but had different safety profiles and impact on hemodynamics leading to differences in country-by-country availability and restrictions in prehospital use.

### Emergency department recommendations categorized as uncertain

Only three controversial statements generated disagreement in the light of new evidence or need for further studies.

2.a.”REBOA can be used for temporary proximal hemorrhage control in the setting of trauma induced resuscitation.”

Resuscitative endovascular balloon occlusion of the aorta (REBOA) is intended as a bridge to definitive hemorrhage control for patients with non-compressible subdiaphragmatic hemorrhages, particularly those who are unresponsive to resuscitation. Notwithstanding heterogenous availability across European countries, there is strong evidence against a standard recommendation for its use in every case, which has been published after the latest version of the S3-Guidelines, generating a strong disagreement in the consensus panel.

Clinical studies have shown no consistent survival benefit [23, 24], or even increased mortality, as highlighted in the UK-REBOA randomized trial [25]. This trial conducted across 16 UK trauma centers, assessed the impact of REBOA on survival in patients with exsanguinating hemorrhage. The findings showed no mortality benefit at 90 days for patients receiving REBOA in addition to standard care compared to those receiving only standard care. In fact, the trial reported a slight increase in mortality rates in the REBOA group (54%) compared to the standard care group (42%) at 90 days. This trend was consistent even at earlier mortality points, suggesting that REBOA might not provide the anticipated survival advantage seen in previous studies. Furthermore, higher mortality from bleeding in the REBOA group, particularly within the first 24 h post-intervention (32% REBOA vs. 17% Non-REBOA group) was found. The trial was terminated early due to harm and provides a critical perspective on the effectiveness of REBOA. While earlier studies demonstrated hemodynamic stability and reduced blood loss, the results of the UK-REBOA trial highlight the need for optimized patient selection and further investigation on the timing of the procedure. These findings suggest that REBOA may not be beneficial for trauma patients and emphasize the importance of robust, high-quality studies to establish clear guidelines and identify the ideal patient population for its use.

Furthermore, it is important to consider the need for training of the trauma team, as unresponsive patients with non-compressible subdiaphragmatic hemorrhage are unusual in Europe. Same is true for its complications, such as vascular access issues, device-related injuries and ischemic complications including compartment syndrome, organ failure and amputations [26, 27].

2.b.” Patients with life-threatening hemorrhage and/or shock, should receive additional administration of fibrinogen (initially 3–6 g or 30–60 mg/kg)”

Fibrinogen is the first coagulation factor critically depleted in acute bleeding. Hypofibrinogenemia is an independent risk factor for shock severity, massive transfusion requirement and mortality [28, 29], and current European guidelines recommend maintaining its plasma level above 1.5 g/dL (GoR 1 C) and suggest an initial fibrinogen supplementation of 3–4 g for trauma patients at risk of massive hemorrhage with a GoR 2b [7]. However, treatment with fibrinogen concentrate (FC) or cryoprecipitate was not associated with a decrease in transfusion requirements or survival benefit [30–32]. The CRYOSTAT- 2 RCT showed that the addition of early and empirical high-dose cryoprecipitate to standard care did not improve all cause 28-day mortality [32]. This study randomized 1604 trauma patients, who required the activation of a major hemorrhage protocol, to the equivalent of 6 g fibrinogen as cryoprecipitate, within 90 min of randomization, in addition to and versus standard treatment in the control group.

There are just a few small RCT for FC application in trauma patients, most of them with the primary aim to test the feasibility of administration and clot strength. The FC dose proposed by the German Guidelines is mainly based on the results and secondary analysis of the RETIC trial, a single center randomized study comparing the use of coagulation factor concentrates (CFC) versus fresh frozen plasma (FFP) as first-line treatment of trauma induced coagulopathy [33, 34]. This trial was terminated after planned interim analysis demonstrated that half of the patients in the FFP group required rescue therapy and 66% developed a multiorgan failure compared to 4% and 50%, respectively, in the CFC group. FFP was insufficient to correct hypofibrinogenemia or significantly improve clot strength compared to CFC in adult trauma patients. Up to 40% of CFC group received FXIII concentrate, leading the authors to recommend early coagulation factor supplementation for severe bleeding.

Empirically-based and viscoelastic assay-guided transfusion protocols coexist in Europe. Both approaches are used in the same country or even in the same institution dependent on the availability of viscoelastic tests and CFC or experience. Examining the existing evidence through the disconjugate lens of heterogenous treatment protocols for trauma induced coagulopathy across Europe, the panel of experts agreed on the recommendation to maintain plasma fibrinogen levels above 1.5 g/dL but did not find appropriate an early supplementation with FC at the proposed dose for all European settings.

2.c. “The pelvis must be physically examined”.

Consensus failure for this recommendation was primarily explained by different practice pattern variability in the Emergency Room clinical assessment of severely blunt injured patients with a pelvis binder applied in the prehospital setting. Complete physical examination of the severely injured patient on arrival to the emergency department is mandatory. Manual compression assessment of pelvic ring stability guides decision-making around mechanical stabilization, but this maneuver should not be repeated to avoid a worsening of a potential deadly bleed.

The low fidelity of clinical examination in the prehospital diagnosis of pelvic fracture has driven empiric pelvic binder application in the field [35, 36]. Consequently, most patients arrive at the emergency department with a pelvic binder, and removal to facilitate clinical examination is often deprioritized in favor of imaging of the pelvis. Disparate local protocols on when and where to release the binder across Europe reflect the difficult balance between avoiding missed pelvic fractures at the CT scan and the risk of recrudescent bleeding with consequent hemodynamic shock [37].

### Limitations

This consensus was developed by 15 European Societies on Trauma and Emergency Surgery, representing 13 countries. Therefore, several European countries did not participate, rendering no opportunity to consider their local peculiarities and organizational models. However, the countries engaged in this project constitute a representative sample of European disparities in terms of geographic, demographic, and economic characteristics, trauma system model and maturation that we believe that the appropriateness of the proposed recommendations can be applied to most of the European settings. When we designed the study, we decided to keep a manageable number of recommendations. Thus, the consensus only included GoR A and GPP recommendations, omitting several aspects of the management of the severely injured patient.

### Future work

The ESTES consensus guidelines on the management of polytrauma represent aggregated evidence-informed expertise readily deployable across European healthcare contexts. AI-assisted utilization of these evidence-informed consensus recommendations, whether via smartphone applications or integrated with electronic health records, offers the potential of real-time, point-of-care decision support for surgeons treating multiply or severely injured patients. Such implementation initiatives stand to reinforce uniform trauma care standards and enhance clinical responsiveness. Future projects might include validation of the appropriateness and implementability across global resource-limited settings.

## Conclusions

Greater than 90% of the evidence- and consensus-based GoR A and GPP recommendations of the S3-guidelines of the DGU are applicable across European healthcare settings. As a result, these ESTES recommendations are the first European guideline that comprehensively supports the provision of trauma treatment from the prehospital setting to the end of the first surgical phase. They serve as a foundational tool for the development of national guidelines, particularly in regions with evolving trauma systems, and promote alignment towards a uniform standard-of-care across Europe.

## Data Availability

No datasets were generated or analysed during the current study.
